# Clinicopathologic Features of Lymphoproliferative Neoplasms Involving the Liver

**DOI:** 10.3390/medicina58010072

**Published:** 2022-01-04

**Authors:** I Chiang, Ren-Ching Wang, Ying-Ching Lai, Chung-Che Chang, Chuan-Han Chen, Chiann-Yi Hsu, Chi-Hung Chen

**Affiliations:** 1 Department of Pathology and Laboratory Medicine, Taichung Veterans General Hospital, Taichung 40705, Taiwan; scidmax@hotmail.com (I.C.); renchingw@gmail.com (R.-C.W.); t20001327@yahoo.com.tw (Y.-C.L.); 2 Department of Nursing, College of Nursing, Hungkuang University, Taichung 40705, Taiwan; 3 Department of Pathology and Laboratory Medicine, Florida Hospital, Orlando, FL 32803, USA; C.Jeff.Chang.MD@adventhealth.com; 4 Department of Radiology, Taichung Veterans General Hospital, Taichung 40705, Taiwan; bonanza0622@gmail.com; 5 Biostatistics Task Force, Taichung Veterans General Hospital, Taichung 40705, Taiwan; chiann@vghtc.gov.tw; 6 Division of Gastroenterology, Department of Internal Medicine, Cheng Ching General Hospital, Taichung 40705, Taiwan

**Keywords:** primary hepatic lymphoma, liver function tests, radiology, pathology, liver

## Abstract

*Background and Objectives*: Primary hepatic lymphoproliferative neoplasms (PHL) are uncommon. This retrospective study is aimed to present the clinicopathological characteristics of PHL and compare to secondary hepatic lymphoproliferative neoplasms (SHL). *Materials and Methods*: Patients who were diagnosed with lymphoproliferative neoplasms involving the liver between January 2004 and December 2018 at a tertiary medical center in central Taiwan were included. The demographic and clinical data, radiological results and histopathological findings were reviewed and summarized. *Results*: We analyzed 36 patients comprising 6 PHL patients and 30 SHL patients. The median age at diagnosis tended to be younger in PHL than in SHL (59 vs. 63 years old, *p* = 0.349). Both entities had a small male predominance. The PHL patients tended to have higher levels of aspartate aminotransferase, alanine transaminase and serum albumin and lower levels of alkaline phosphatase, total bilirubin, γ-glutamyl transferase and lactate dehydrogenase compared with SHL, but there was no significant difference. Multiple mass lesions were the most common radiological finding in both groups. Diffuse large B-cell lymphoma was the predominant subtype in both groups (67% in PHL and 40% in SHL). The PHL patients had a longer median survival than the SHL patients (not reached vs. 3 months, *p* = 0.003). *Conclusions*: Although there was no significant difference between PHL and SHL in clinical, laboratory and radiological features, the SHL patients had very poor outcomes with a median survival time of 3 months. Effective therapies are urgently required for these patients.

## 1. Introduction

Lymphoproliferative neoplasms involving the liver are rare conditions [1,2]. The majority of liver lymphoproliferative neoplasms are a secondary involvement of systemic disorders and are classified as stage IV disease. In Asian and Western countries alike, primary hepatic lymphoproliferative neoplasms (PHL) constitute <1% of the extra-nodal lymphomas [2,3]. The clinical presentation of hepatic lymphoproliferative neoplasms is usually non-specific. Elevated levels in liver function tests, including liver enzymes, bilirubin and lactate dehydrogenase (LDH), are noted in over half of the cases with PHL [4]. Hepatic function abnormalities are driven by mechanisms such as the following: tumor infiltration in the liver parenchyma, obstructive jaundice due to massive enlarged lymph nodes or mass lesions, paraneoplastic syndromes, sinusoidal infiltration of histiocytes with hemophagocytosis, infection or reactivation of viral hepatitis due to treatment [5]. Liver biopsy is helpful to determine the exact cause of liver function abnormality in this group of patients. 

PHL are usually presented as hepatic mass lesions [1,3,4,6] mimicking hepatocellular carcinoma or metastatic carcinoma. Accurate pre-management diagnosis is fundamental for choosing the best treatment. However, an accurate diagnosis of hepatic hematologic neoplasms based on needle core biopsy is often challenging due to the limited tumor cells and concurrent inflammatory reactions [7]. Hepatic lymphoma may show nodular/mass, portal infiltration and sinusoidal histologic patterns in liver, and various histological subtypes of lymphoma are known to associate with these histologic patterns [3].

To the best of our knowledge, clinicopathologic features distinguishing PHL and secondary hepatic lymphoproliferative neoplasms (SHL) are not fully characterized. To define these features, we reviewed those cases with biopsy-proven lymphoproliferative neoplasms, either primary or secondary, from a tertiary medical center in Taiwan in this retrospective study.

## 2. Materials and Methods

### 2.1. Patients

We extracted cases of lymphoproliferative neoplasms with hepatic involvement, either primary or secondary, that had been diagnosed with pathological evidence at a tertiary medical center in central Taiwan between January 2004 and December 2018. Diagnostic specimens were collected either from needle biopsies or resected liver specimens. 

Cases were subdivided into two groups. The first group was PHL, according to criteria proposed by Lei. [2]. The diagnosis of PHL fulfilled the following criteria at the time of presentation: (a) showing symptoms caused by liver involvement; (b) the absence of distant lymphadenopathy (not palpable clinically and/or not detected during staging radiologic studies); and (c) the absence of a leukemic blood picture in the peripheral blood film. The second group was SHL, with either a previous history of a lymphoproliferative neoplasm or concurrent extra-hepatic involvement at presentation. A liver biopsy of the SHL group may be obtained before treatment or at recurrence.

Most patients were treated with chemotherapy, radiotherapy and/or surgery based on the standard of care and clinical condition except one patient who had expired before diagnosis. The follow-up data were collected until July 2019. This was a retrospective study, and the data were obtained from de-identified information of all cases, and thus informed consent from patients was not needed based on the institutional policy. This study was approved by the institutional review board (IRB: CE20060A).

Clinical data included the following: demographic details, international prognostic index (IPI) score, viral infection statuses of hepatitis B (HBV), hepatitis C (HCV) and human immunodeficiency (HIV), hemoglobin levels, liver function tests, findings of ultrasonography and computed tomography (CT) and magnetic resonance imaging (MRI). These data were retrieved from electronic medical records of patients. The IPI score was the sum of the numbers of the risk factors: age >60 years, raised LDH, stage III or IV of disease, performance status ≥2 and more than 1 extra-nodal site of disease. Results of image studies (CT, ultrasonography and/or MRI) were classified as either “mass lesion” or “non-mass lesion” based on the presence/absence of visible mass lesions. Among cases with “non-mass lesion”, those showing no hepatomegaly were considered as negative findings.

We reviewed study results of liver biopsy, diagnostic immunohistochemistry (IHC) and Epstein–Barr encoding region (EBER) in situ hybridization for the Epstein–Barr virus (EBV). Lymphoproliferative neoplasms were classified according to the 2017 World Health Organization (WHO) classification [8]. Three histological patterns in the liver were described as follows: (a) “nodular/mass” pattern with presence of sizable aggregation of tumor cells, (b) “sinusoidal pattern” with tumor cells infiltrated into sinusoidal space of liver and (c) “portal infiltrative pattern” with tumor cells involved in the portal area without sizable mass lesions.

### 2.2. Statistical Analysis

To assess inter-group differences, we applied the Chi-square test, Fisher’s exact test and Mann–Whitney U test. Statistical significance was set at *p* < 0.05. The Kaplan–Meier method was used to estimate the observed survival times, and the log rank test was used to compare the two groups. The follow-up time was measured from the date of diagnosis of liver disease to the date of death. Analyses were performed using the Statistical Package for the Social Science (IBM SPSS version 22.0; International Business Machines Corp, New York, NY, USA).

## 3. Results

### 3.1. Demographic Details, Clinical Features and Laboratory Findings

Forty-two patients with lymphoproliferative neoplasms involving the liver were identified from the archive, and three of them were excluded due to insufficient specimens for analysis or incomplete clinical data. Three cases diagnosed as inflammatory pseudotumor-like follicular dendritic cell sarcoma were also excluded. Thirty-six cases were finally analyzed. Eighteen cases initially presented with a hepatic lymphoproliferative neoplasm had concurrent extra-hepatic involvement, 12 cases had a previous history of extrahepatic lymphoma, and they were classified as SHL. The remaining six cases were classified as PHL. The diagnosis of PHL was confirmed by a liver biopsy and subsequent image surveys, either by a CT or positron emission tomography scan, to rule out extra-hepatic lymphoma. Together, they included 20 cases of mature B cell lymphoma, 12 cases of mature T cells lymphoma, 3 cases of classical Hodgkin lymphoma and 1 case of EBV-associated polymorphic post-transplant lymphoproliferative disorder (PTLD) (Table 1). 

Diffuse large B-cell lymphoma (DLBCL) was the most prevalent subtype in both the PHL (67%) and SHL (40%) groups. Only one case (17%) in the PHL group, diagnosed as hepatosplenic T cell lymphoma (HSTCL), involved bone marrow. In contrast, 17 cases (61%) in the SHL group had bone marrow involvement (out of the 28 cases with available bone marrow specimens). However, there was no statistical difference between both groups in bone marrow involvement (*p* = 0.078).

The mean age at diagnosis tended to be younger in the PHL cases as compared with SHL (58.1 vs. 59.52 years old, *p* = 0.349, Table 2). Male predominance (the male-to-female ratio is 2:1 in PHL and 1.73:1 in SHL) was found in both groups. The SHL patients frequently presented with B symptoms at initial diagnosis (50% in PHL and 73% in SHL, *p* = 0.343). Only two patients in the SHL group had histories of autoimmune diseases (rheumatic arthritis and systemic lupus erythematosus). Concurrent viral infections of one of HBV, HCV, HIV (indicated by virology test and/or clinical history) and EBV (by EBER on biopsy specimens) were found in 2 PHL and 16 SHL cases (data not shown). There was no intergroup difference regarding these viral infection rates.

The SHL patients tended to have lower hemoglobin levels (113.8 g/L in PHL and 98.4 g/L in SHL, *p* = 0.199), and they also had significantly lower platelet counts (266 × 10^9^/L in PHL and 130.87 × 10^9^/L in SHL, *p* = 0.024). Together with decreased levels of hemoglobin, platelet count and serum albumin, increased levels of the following tests were noted in both groups: aspartate aminotransferase (AST), alanine aminotransferase (ALT), LDH, alkaline phosphatase (ALP), γ-glutamyl transferase (GGT) and total bilirubin (Table 2). Of note, the PHL patients tended to have higher levels of AST, ALT and serum albumin and lower levels of ALP, total bilirubin, GGT and LDH compared with SHL; however, the intergroup differences were statistically insignificant. In both groups, solid tumor markers, carcinoembryonic antigen (CEA) and alpha-fetoprotein (AFP) were all within the normal limits, without intergroup difference.

### 3.2. Image Studies

**Figure 1:**
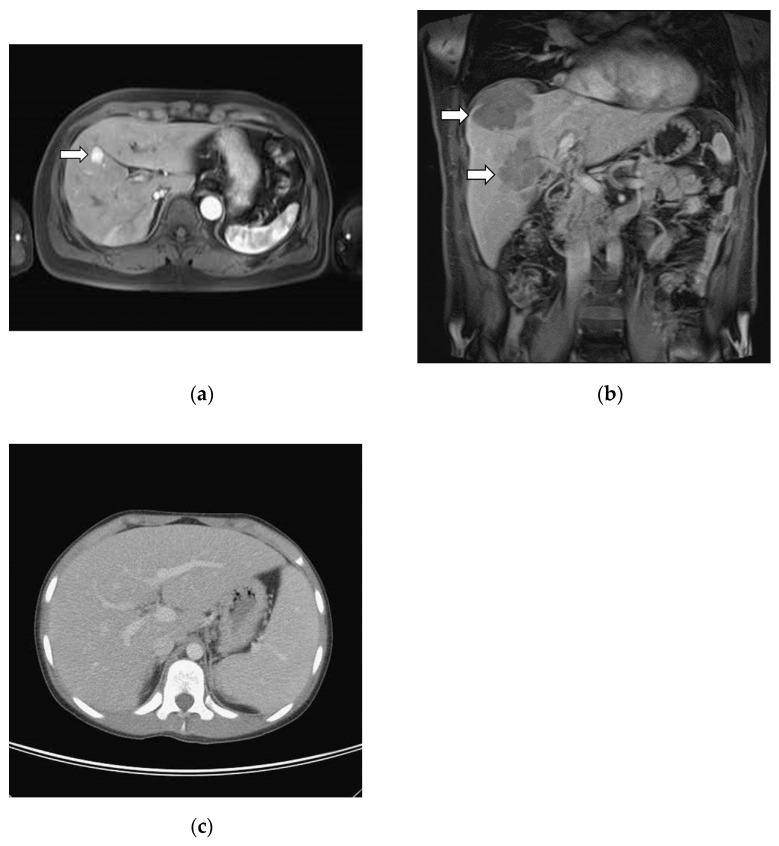
Variable radiographic findings in primary hepatic lymphoma: (**a**) Abdominal magnetic resonance imaging (MRI) axial T1-weighted fat-saturated image with contrast enhancement, arterial phase. MAGNETOM SymphonyTim 1.5T MRI scanner (Siemens, Erlangen, Germany). This case underwent segmental resection and final diagnosis is primary liver low grade follicular lymphoma (Figure 2a,b). Patient is still alive without evidence of disease 7 years after initial diagnosis; (**b**) Abdominal MRI coronal T1-weighted fat-saturated image with contrast enhancement, venous phase, from 55-year-old male. MAGNETOM Aera 1.5T MRI scanner (Siemens, Erlangen, Germany). Multiple liver mass lesions (arrow) were found but no other lymphadenopathy. Biopsy of liver showed diffuse large B cell lymphoma, germinal center B phenotype. Patient was treated with R-CHOP in partial response and was alive 3 years after initial diagnosis; (**c**) Abdominal computed tomography axial image with contrast enhancement, venous phase. Brilliance 64 CT scanner (Philips, Cleveland, OH, USA). Apparent hepatomegaly and splenomegaly noted from 12-year-old body. Random liver biopsy had sinusoidal atypical T lymphocytes infiltration and clonality studies for TCR-gamma was clonal. Final diagnosis is hepatosplenic T cell lymphoma. Patient is alive without disease 8 years after initial diagnosis.

Radiographic studies from 5 cases (5/6, 88.9%) in the PHL group and 21 cases (21/29, 72.4%) in the SHL group showed mass-forming lesions (Table 3), and multiple hepatic masses were commonly observed (3/6, 50% in PHL vs. 19/29, 66% in SHL) Of interest, hepatic malignancy was the suspected diagnosis in only 40% of the PHL patients and 24% of the SHL patients with mass-forming lesions. Figure 1a shows radiographic features of a case of primary liver follicular lymphoma with a single mass-forming lesion, and Figure 1b, a case of primary hepatic DLBCL with multiple mass lesions.

Among the nine patients without radiological evidence of mass-forming lesions (one case in PHL and eight cases in SHL), four of them had hepatomegaly, and five showed negative image findings. The only PHL case without a mass forming lesion was a patient with HSTCL. He presented clinically with acute hepatitis and no significant radiologic finding was found with his liver (Figure 1c). A liver biopsy showed small lymphocytes infiltrations in the sinusoidal space. All the cases of negative radiologic findings had abnormal liver function tests with elevated serum levels of either one of the following: AST, ALT, ALP, LDH and with an increased total bilirubin.

### 3.3. Correlation between Radiographic Findings and Histological Patterns

**Figure 2:**
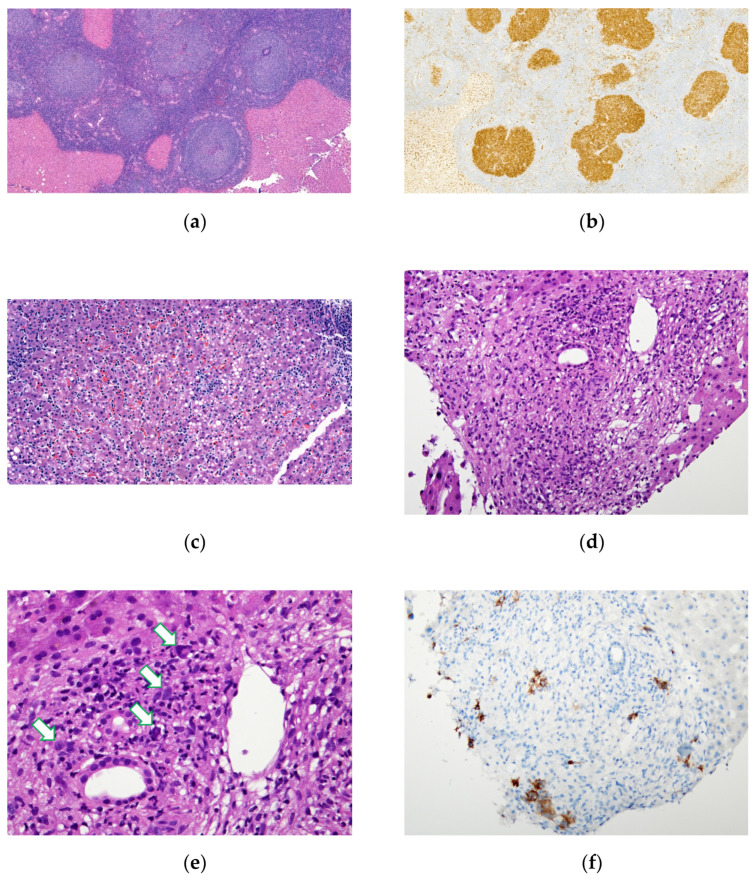
Variable pathologic findings in primary liver lymphoma: (**a**,**b**) 65-year-old male with primary hepatic follicular lymphoma, same patient in Figure 1a. (**a**) Hematoxylin and Eosin (HE) stain, ×40 magnification. Resected liver parenchyma showed nodular distribution of atypical lymphoid follicles with attenuated mantle zone and loss of polarity in germinal center. (**b**) Immunohistochemistry (IHC) stain for CD10, ×40 magnification. Strong-stained pattern in germinal center. (**c**) 12-year-old male with hepatosplenic T-cell lymphoma, same patient in Figure 1c. Liver needle biopsy showed sinusoidal infiltration of atypical lymphocytes. HE stain, ×100 magnification. (**d**–**f**) 80-year-old male patient with secondary hepatic involvement with classical Hodgkin lymphoma. (**d**) HE stain, ×100 magnification. Needle biopsy of liver showed ill-formed granuloma over portal area. (**e**) Mummified Hodgkin cells (arrow) were barely noted in high power and masked by inflammatory cells. HE stain, ×400 magnification. (**f**) More Hodgkin cells are highlighted by CD30 IHC stain, ×100 magnification.

Nodular/mass, portal and sinusoid patterns were identified histologically. Pure nodular pattern is the most prevalent histologic pattern in B-cell lymphoma patients with radiographic mass-forming lesions in either the PHL or SHL groups. Figure 2a,b shows an example of follicular lymphoma with the nodular pattern. On the other hand, a mixed histologic pattern was predominant in the cases of T-cell lymphoma. 

A histological diagnosis of mass-forming lesions was typically rather straightforward, and the diagnosis was confirmed with immunohistochemistry.

Five cases had negative image findings, neither a mass-forming lesion nor hepatomegaly was noted, including one case of HSTCL in the PHL group, and one case of DLBLC, two cases of T cell lymphoma and one case of EBV-positive polymorphic PTLD in the SHL group. The diagnosis of these lesions was more challenging and required thorough morphologic and immunophenotyping evaluations. A liver biopsy from a patient with negative image findings revealed either the sinusoidal pattern or small discrete nodules involving the portal area. Figure 2c shows an example of the sinusoidal pattern of HSTCL in a patient who also had no significant image findings.

Tumor cells were sometimes masked by the background, especially in the case of Hodgkin lymphoma (Figure 2d,e). In this situation, mummified Hodgkin cells were difficult to recognize without IHC staining for CD30 (Figure 2f).

### 3.4. Prognosis and Clinical Outcomes

All the SHL patients had stage IV disease. Most of the PHL patients had stage I disease, with the exception of a case of stage IV disease diagnosis of HSTCL in a patient who subsequently developed bone marrow involvement. The median follow-up period was 46.9 months (range: 8.1–85.9 months) and 3 months (range: 0.1–26.5 months) for the PHL and SHL patients, respectively. No evidence of subsequent nodal disease was found during the follow-up period in the PHL group. The IPI scores in the SHL patients were significantly higher than in the PHL patients (*p* < 0.001, Table 2). For those diagnosed with DLBCL, 11 of the 12 SHL cases had high risk IPI scores (≥4), and all four PHL cases had IPI scores <4. The complete response was much higher for cases with PHL than with SHL (5/6, 83.3% vs. 2/25, 8%, *p* < 0.001). Similarly, the overall survival was better for the patients with PHL than with SHL (median survival: not reached, vs. 3 months, *p* = 0.003). The SHL patients had exceedingly poor prognosis, as 22 patients (88%) had died of the disease, mostly <5 months after diagnosis of the liver biopsy.

## 4. Discussion

Lymphoproliferative neoplasms of the liver constitute <1% of all liver malignancies [2,3,9], and most of them are secondary involvement [1,4,10], representing an advanced stage of lymphoproliferative disorders. PHL, defined as a liver-confined disease without evidence of extra-hepatic tissue involvement within 6 months of initial diagnosis [2,11], constitute <1% of all lymphoproliferative neoplasms [2,12,13]. DLBCL was reported to be the most common type in both the PHL and SHL groups [2,4,5,6], which was also consistent with our results. 

Our findings revealed several distinguishing clinicopathological features between PHL and SHL. The median age of SHL patients tended to be older than PHL patients (63 years versus 59 years). This agrees with the previous reports showing that the mean age of PHL patients is usually in their fifth to sixth decades [2,4,13], and the SHL patients are older (>60 years old) [5]. 

We found that patients of both groups presented with abnormalities of liver function tests, including elevation in serum AST, ALT, ALP, total bilirubin, GGT and LDH. These findings are similar to previous reports [4,14]. Our study showed no statical difference between both groups, suggesting that biomarkers alone cannot be used for differentiating between PHL and SHL clinically. All our patients showed no elevation of CEA and AFP, and such features might be helpful for a differential diagnosis [5,9,14,15]. Of importance, abnormal liver function tests were observed in all nine patients without mass-forming lesions. Hence, a liver biopsy is still recommended for patients with unexplained abnormal liver function tests and inconclusive image findings. 

Radiologically, mass-forming lesions are the most common presentation in hepatic lymphomas in the literature [5] and similar results were found in both groups of our patients. Our results further indicated that both PHL and SHL can present as multiple hepatic masses. Of note, mass-forming lesions with ring-like enhancement in dynamic CT and MRI [5,16,17,18], and vascular penetration in the mass-forming lesion, have been reported to be specific radiologic features in PHL cases [9,15,19]. However, such radiologic features overlap with other hepatic tumors and are, therefore, nonspecific [15,18]. The majority of our cases (60% of patients with PHL and 76% of patients with SHL) with radiological mass-forming lesions were initially suspected to have benign lesions or malignancy other than lymphomas. For a patient with multiple masses in their liver image, not only metastasis but lymphoproliferative neoplasm should be included in their differential diagnoses.

Nodular, sinusoidal and portal histological patterns have been documented in hepatic lymphoma with an association with variable lymphoma subtypes [3]. The histologic pattern is usually correlated with the radiologic presentation [10,15]. In the present study, liver lymphomas with the histologic nodular pattern typically showed mass-forming lesions, while lymphomas with histologic sinusoidal or portal patterns usually presented as hepatomegaly. For example, follicular lymphoma typically showed a nodular pattern with mass-forming lesions (Figure 1a), and B lymphoblastic leukemia/lymphoma and HSTCL typically presented as a sinusoidal pattern associated with hepatomegaly (Figure 1c) [13]. In our study, the nodular pattern had the most common occurrence in the B-cell lymphoma of both groups. Our study showed that the radiological presentation of multiple hepatic masses was common in hepatic lymphoproliferative neoplasm. In this situation, differentiating hepatic mass lesions between lymphomas and metastatic solid cancers would be very difficult without biopsy evidence. 

Diagnosing lymphoma with secondary liver involvement can be challenging, especially for cases with portal or sinusoidal infiltrations. Liver involvement in the case of Hodgkin lymphoma with discrete portal nodules is difficult to recognize mummified Reed–Sternberg cells or Hodgkin cells without immunohistochemistry [7]. A definitive diagnosis may require multiple percutaneous needle biopsies or larger wedge-resected specimens [1]. Sinusoidal infiltrations of T lymphocytes are also nonspecific for T cell lymphoma. EBV-induced infectious mononucleosis and hepatitis also show a similar pattern of infiltration in a liver biopsy [20], making a differential diagnosis between HSTCL and EBV-associated hepatitis difficult. Findings of an extensive cellular atypia, bone marrow involvement, less prominent portal tract involvement and a sinusoidal infiltrate of CD56+ T cells should raise suspicion on the diagnosis of HSTCL. 

DLBCL was the majority of both groups (67% in PHL and 40% in SHL). Both groups of DLBCL patients had male predominance and commonly showed multiple mass lesions in the liver radiologically and a nodular histologic pattern. The DLBCL patients in the PHL group had a younger age; higher levels of ALT, total bilirubin, GGT, serum albumin and platelet count; and lower levels of hemoglobin, AST, ALP and LDH. However, the above features had no statistical difference between PHL and SHL in the DLBCL patients.

The hepatic involvement of lymphoproliferative neoplasms is often considered as stage IV disease with poor prognosis. However, hepatic involvement alone may not qualify in the current Lugano staging system. Taking the situation of DLBCL, the most common type of lymphoma in both groups, our PHL patients had lower IPI scores and with much better prognosis than the SHL patients. This is consistent with the previous studies [2,6,12]. Bone marrow involvement is also less common in PHL than in SHL. In PHL patients, complete remission for chemotherapy was reported to be as high as 83% in the literature [4,5,6,13,18,21]. Surgical resection combined with adjuvant chemotherapy could be applied on selective PHL patients, who are young and have localized, and single lymphoma without liver involvement of the right lobe [17,18].

Our SHL patients had a particularly poor prognosis with a median survival of 3 months and most of them died of disease within 5 months of diagnosis. These findings are similar to a previous study by Kumar et al. [22]. Comprehensive staging (imaging studies and bone marrow examinations) is essential for diagnosing these patients. Additionally, developing novel therapies are needed to improve the outcomes of these patients.

The small size of the studied population and the retrospective design are the main limitations of our study. The limited number of cases result in representative bias, and complete laboratory and radiological data cannot be obtained in every case due to the different clinical situation at that time. However, our study observed some differences between the PHL and SHL groups, and a larger study with more cases is needed to verify these observations.

## 5. Conclusions

Although PHL and SHL share a number of similar clinicopathological characteristics, prognosis between the two groups is different. Comprehensive staging, including radiological studies and bone marrow examinations, is essential for the precise prediction of prognosis and formulating appropriate treatment plans for patients with hepatic lymphomas.

## Figures and Tables

**Table 1:** Pathologic classification of hepatic lymphoma.

Pathologic Classification ^a^	PHL (*n* = 6)		SHL (*n* = 30)	
**Mature B cell lymphoma**	**5**	**(83%)**	**15**	**(50%)**
Diffuse Large B-cell Lymphoma	4	(67%)	12	(40%)
Burkitt’s Lymphoma	0		2	(7%)
Follicular Lymphoma	1	(17%)	0	
Mature B-cell Lymphoma (Unclassified due to limited tissue)	0		1	(3%)
**Mature T cell lymphoma**	**1**	**(17%)**	**11**	**(37%)**
Peripheral T-cell lymphoma	0		5	(17%)
NK/T-cell lymphoma, nasal type	0		1	(3%)
Anaplastic Large-cell Lymphoma	0		1	(3%)
Hepatosplenic T cell lymphoma	1	(17%)	0	
Mature T-cell Lymphoma (Unclassified due to limited tissue)	0		4	(13.3%)
**Hodgkin lymphoma**	**0**	**(0%)**	**3**	**(10%)**
Classical Hodgkin Lymphoma				
**Other**	**0**	**(0%)**	**1**	**(3%)**
EBV-associated polymorphic posttransplant lymphoproliferative disorder	0		1	(3%)

Abbreviations: PHL, primary hepatic lymphoma; SHL, secondary hepatic lymphoma; EBV, Epstein–Barr virus. ^a^ Chi-square test. Fisher’s exact test. A *p*-value less than 0.05 is statistically significant. No significant difference (*p* = 0.494) between both groups in composition of disease. Categorical data were expressed as number and percentage.

**Table 2:** Demographic and Laboratory findings.

	PHL (*n* = 6)			SHL (*n* = 30)			*p* Value
	N ^#^			N ^#^			
**Age at Diagnosis**^a^ (years) Mean ± SD (median)	6	58.1	±17.36 (59)	30	59.52	±16.57 (63)	0.349
**Sex** ^a^				30			1.000
Male		4	(66.67%)		19	(63.33%)	
Female		2	(33.33%)		11	(36.67%)	
**B symptom** ^a^	6	3	(50%)	30	22	(73.33%)	0.343
**IPI score** ^a^	6			29			<0.001
0–1		1	(16.67%)		0		
2–3		5	(83.33%)		5	(17.24%)	
4–5		0			24	(82.76%)	
**Lab**^a^ (Mean ± SD)							
Hemoglobin (g/L)	6	113.8	±23.8	30	98.4	±18.7	0.149
Platelet (×10^9^/L)	6	266	±57.94	30	130.87	±89.09	<0.001
AST (U/L)	6	192.17	±229.92	30	146.13	±139.04	0.893
ALT (U/L)	6	217.46	±91.53	30	91.53	±90.75	0.246
ALK-P (U/L)	6	499.33	±566.57	30	637.43	±688.85	0.268
Total Bilirubin (μmol/L)	6	76.11	±131.7	29	78.49	±98.5	0.542
Direct Bilirubin (μmol/L)	4	65	±96.47	27	48.56	±64.3	0.898
LDH (U/L)	6	991	±610.89	29	1135.14	±1691.41	0.589
GGT (U/L)	2	329	±173.95	10	518.50	±461.60	0.606
Albumin (g/L)	6	32.8	±6.6	29	29.6	±6.6	0.372
AFP (μg/L)	3	3.6	±0.86	15	3.47	±2.71	0.426
CEA (μg/L)	1	2.45		13	3.32	±2.29	0.857
**Status of Last Follow-up** ^a^	6			29			
Expired		0	(0%)		21	(72.41%)	0.002
Follow-up (months) (Mean ± SD)		44.52	±28.94		9.46	±14.12	0.002

Abbreviations: PHL, primary hepatic lymphoma; SHL, secondary hepatic lymphoma; SD, standard deviation; IPI, international prognostic index; AST, aspartate aminotransferase; ALT, alanine transaminase; ALK-P, alkaline phosphatase; LDH, lactate dehydrogenase; GGT, γ-glutamyl transferase; AFP, alpha-fetoprotein; CEA, carcinoembryonic antigen. Normal range of lab tests: Hemoglobin: 120.0–175.0 (g/L), Platelet: 150–400 (×10^9^/L), AST: 8–38 (U/L), ALT: 10–50 (U/L), ALK-P: 50–190 (U/L), Total bilirubin: 3.42–20.52 (μmol/L), Direct bilirubin: ≤3.42 (μmol/L), LDH: 120–240 (U/L), GGT: 4–63 (U/L), Albumin: 35.0–50.0 (g/L), AFP: ≤7 (μg/L), CEA: <5 (μg/L). ^a^ Chi-square test. Fisher’s exact test. Mann–Whitney U test. A *p*-value less than 0.05 is statistically significant. Continuous data were expressed as Mean ± SD. ^#^ Numbers of patients with available data.

**Table 3:** Radiographic presentation in both groups.

Image Findings ^a^	PHL	(*n* = 6)	SHL	(*n* = 29 ^#^)
Mass lesion				
Single mass	33%	(2/6)	7%	(2/29)
Multiple masses	50%	(3/6)	66%	(19/29)
Non-mass lesion				
Hepatomegaly	0%	0	14%	(4/29)
Negative image findings	17%	(1/6)	14%	(4/29)

Abbreviations: PHL, primary hepatic lymphoma; SHL, secondary hepatic lymphoma. Categorical data were expressed as number and percentage. ^a^ Chi-square test. Fisher’s exact test. A *p*-value less than 0.05 is statistically significant. No significant difference (*p* = 1) between both groups in composition of disease. ^#^ One case in SHL had no available liver image studies.

## Data Availability

The data presented in this study are available on request from the corresponding author.
